# Artificial intelligence in peptide cancer vaccine design: from neoantigen discovery to immunogenicity prediction

**DOI:** 10.3389/fgene.2026.1875066

**Published:** 2026-07-29

**Authors:** Petar Brlek, Jan Kolić, Luka Bulić, Vedrana Škaro, Dragan Primorac

**Affiliations:** 1 St. Catherine Specialty Hospital, Zagreb, Croatia; 2 International Center for Applied Biological Research, Zagreb, Croatia; 3 School of Medicine, Josip Juraj Strossmayer University of Osijek, Osijek, Croatia; 4 Department of Molecular Biology, Faculty of Science, University of Zagreb, Zagreb, Croatia; 5 Department of Oncology and Radiotherapy, University Hospital Center Zagreb, Zagreb, Croatia; 6 Algebra Bernays University, Zagreb, Croatia; 7 Faculty of Dental Medicine and Health, Josip Juraj Strossmayer University of Osijek, Osijek, Croatia; 8 Eberly College of Science, The Pennsylvania State University, State College, PA, United States; 9 School of Medicine, University of Split, Split, Croatia; 10 The Henry C. Lee College of Criminal Justice and Forensic Sciences, University of New Haven, New Haven, CT, United States; 11 Sana Kliniken Oberfranken, Coburg, Germany; 12 School of Medicine, University of Rijeka, Rijeka, Croatia; 13 School of Medicine, University of Mostar, Mostar, Bosnia and Herzegovina; 14 National Forensic Sciences University, Gandhinagar, India; 15 School of Medicine, University of Pittsburgh, Pittsburgh, PA, United States

**Keywords:** artificial intelligence, cancer immunotherapy, HLA binding, immunogenicity prediction, immunopeptidomics, neoantigens, peptide cancer vaccines, precision oncology

## Abstract

Peptide-based cancer vaccines represent a promising immunotherapeutic strategy aimed at inducing tumor-specific immune responses through the targeting of tumor-associated antigens and neoantigens. Recent advances in next-generation sequencing and immunogenomics have accelerated the identification of candidate neoantigens; however, the development of effective peptide vaccines remains limited by challenges related to antigen selection, HLA polymorphism, antigen processing, and variability in immunogenicity. Artificial intelligence (AI), including machine learning and deep learning approaches, has emerged as a transformative tool capable of addressing these limitations through large-scale integration and analysis of genomic, transcriptomic, proteomic, and immunological data. In this review, we summarize the current role of AI across the peptide cancer vaccine development pipeline, from neoantigen discovery and epitope prioritization to prediction of peptide–HLA binding, antigen presentation, and T-cell receptor recognition. We discuss the application of modern computational frameworks, including pan-allelic prediction models, transformer-based architectures, immunopeptidomics-informed learning, and multi-modal AI systems integrating tumor and immune microenvironment data. Furthermore, we examine the clinical translation of personalized neoantigen vaccines, including their combination with immune checkpoint inhibitors and their emerging role in aggressive malignancies such as glioblastoma. Despite substantial progress, significant challenges remain, including high false-positive prediction rates, limited diversity of training datasets, biological complexity of immunogenicity, and regulatory and manufacturing barriers associated with individualized therapies. Continued integration of AI-driven prediction tools with experimental validation and translational immunology will be essential for the development of clinically effective and scalable precision cancer vaccines.

## 1 Introduction and immunological background

Cancer remains a major global health challenge and a leading cause of death worldwide (The global challenge of cancer, 2020). Advances in high-throughput sequencing have enabled comprehensive characterization of tumor biology, providing critical insights into actionable mutations and tumor–immune interactions that inform precision oncology (Brlek et al., 2025). Despite progress in early detection and targeted therapy, several malignancies, including glioblastoma and other aggressive tumor types, remain poorly responsive to conventional treatment (Rezk et al., 2025; Brlek et al., 2021), motivating the development of immunotherapeutic strategies that engage the patient’s own immune system against tumor cells. Among these, cancer vaccines have emerged as a promising modality designed to stimulate tumor-specific immune responses.

Cancer vaccines represent a form of therapeutic immunotherapy that aims to induce or amplify tumor-specific immune responses (Ism et al., 2025). Unlike prophylactic vaccines used against infectious diseases, these vaccines are designed to enhance anti-tumor immunity in patients with established malignancies. Several platforms have been developed, including dendritic cell vaccines, viral vectors, nucleic acid vaccines and whole-cell approaches (Ism et al., 2025; Lee et al., 2023). Among them, peptide-based vaccines remain widely used because of their safety, relatively simple production and ability to target defined antigens. Synthetic peptides derived from tumor antigens can be administered with adjuvants to induce antigen-specific T-cell responses in a controlled and cost-effective manner (Chen X. et al., 2020). The rationale for peptide-based vaccines is grounded in the ability of T-cells to recognize tumor antigens. These antigens can be broadly classified as tumor-associated antigens and tumor-specific neoantigens (Abd-Aziz and Poh, 2022). Tumor-associated antigens are proteins that are overexpressed or aberrantly expressed in malignant cells but can also be present in certain normal tissues. Because they originate from self-proteins, their immunogenicity is often limited by central tolerance (Feng et al., 2025). In contrast, neoantigens arise from somatic mutations that are unique to tumor cells and are absent from normal tissues. As a result, they are not subject to central tolerance and are generally more immunogenic, making them attractive targets for immunotherapy (Xie et al., 2023). Tumor antigen presentation to T-cells is mediated by major histocompatibility complex molecules, referred to as human leukocyte antigens in humans. MHC class I molecules present peptides derived from intracellular proteins to CD8-positive cytotoxic T-lymphocytes (Xie et al., 2023; Wang et al., 2021). These peptides are typically 8 to 11 amino acids in length, generated by proteasomal degradation and transported into the endoplasmic reticulum by the transporter associated with antigen processing (Lee et al., 2024). Recognition of peptide–MHC complexes activates CD8-positive T-cells, enabling direct tumor cell killing through perforin- and granzyme-mediated mechanisms (Todaro et al., 2023). MHC class II molecules are primarily expressed on professional antigen-presenting cells such as dendritic cells, macrophages and B cells, and present peptides derived from extracellular proteins to CD4-positive helper T-cells (Zhu, 2025). These peptides are generally longer, ranging from 13 to 25 amino acids, and are generated in endosomal compartments (Todaro et al., 2023). CD4-positive T-cells play a central role in anti-tumor immunity by promoting dendritic cell activation, supporting CD8-positive T-cell responses and facilitating antibody production. Effective peptide-based vaccines therefore aim to engage both CD8-positive and CD4-positive T-cell populations to achieve robust and durable immune responses (Wang et al., 2021; Todaro et al., 2023; Topchyan et al., 2023).

Despite this strong biological rationale, peptide-based vaccines face several important limitations. Short peptides are often weakly immunogenic and require appropriate adjuvants or delivery systems (Todaro et al., 2023; Jahantigh et al., 2026). Tumor heterogeneity further complicates therapeutic efficacy, as genetically diverse tumor cell populations may not uniformly express the targeted antigen. In addition, extensive polymorphism of human leukocyte antigen genes presents a significant challenge for vaccine design, since different alleles bind distinct peptide motifs, limiting the effectiveness of a single peptide across diverse populations (Villanueva-Flores et al., 2025). These limitations have driven the integration of artificial intelligence into vaccine development. Machine learning approaches enable large-scale analysis of genomic and immunological data, allowing prediction of peptide–MHC binding, identification of immunogenic epitopes and prioritization of clinically relevant neoantigens (Villanueva-Flores et al., 2025; Bulić et al., 2026). By improving antigen selection and supporting personalized strategies, artificial intelligence is shifting peptide-based cancer vaccine development from an empirical approach toward a data-driven framework.

Several recent reviews have surveyed AI applications in epitope prediction and cancer immunotherapy, including comparative analyses of prediction tools (Villanueva-Flores et al., 2025) and broader overviews of AI in precision oncology (Bulić et al., 2026). The present review is positioned differently in three respects. First, rather than cataloguing prediction tools in isolation, we organize the AI-enabled vaccine pipeline as a sequential cascade of biological filters, including expression, processing, presentation, recognition, and effector function, and use this cascade to localize where current models succeed and where they systematically fail. Second, we anchor the discussion in the persistent validation gap exemplified by the TESLA benchmark, in which only 6% of computationally prioritized peptides proved immunogenic, and use this gap as a lens through which to examine the biological determinants that current AI architectures do not yet capture. Third, we connect computational developments to an emerging translational case in glioblastoma, where personalized neoantigen vaccination has recently produced clinically meaningful signals in a setting historically resistant to immunotherapy. Our aim is therefore not to re-enumerate tools but to provide a mechanistically grounded synthesis of what AI currently delivers, where it fails, and what biological constraints will shape its near-term clinical utility.

**FIGURE 1:**
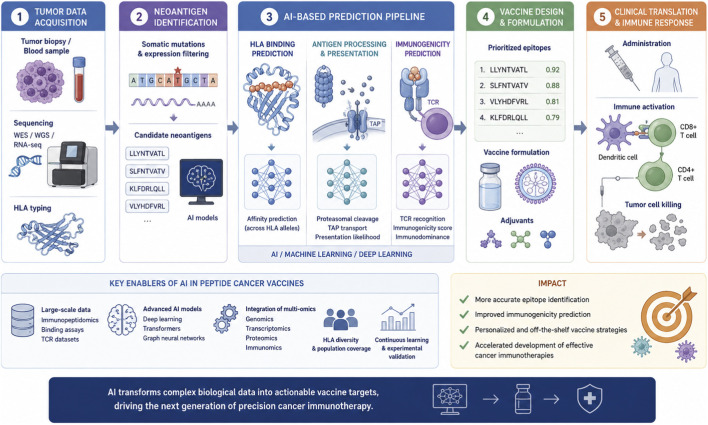
Overview of artificial intelligence applications in cancer vaccine design.

In this review, we provide a comprehensive analysis of how artificial intelligence can be leveraged to improve the design and development of personalized cancer vaccines (Figure 1).

## 2 AI-enabled neoantigen and epitope discovery

**FIGURE 2:**
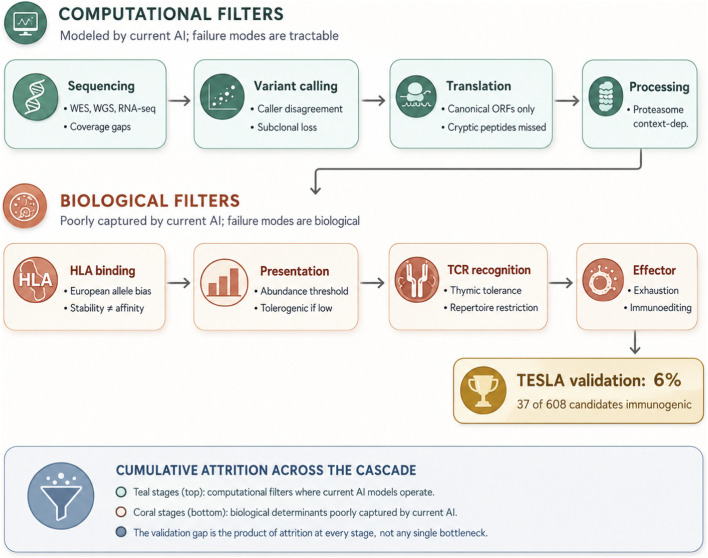
The neoantigen prediction cascade and its failure modes.

A central challenge in the development of peptide-based cancer vaccines is the identification of suitable neoantigens and epitopes capable of eliciting effective immune responses against tumor cells. Tumors harbor a large number of mutations, yet only a small subset yields peptides that (i) are expressed at sufficient levels, (ii) are processed into appropriate peptide fragments, (iii) bind patient-specific HLA molecules, (iv) are presented on the cell surface, (v) are recognized by T-cells, and (vi) produce strong and long-lasting immune reactions (Cai et al., 2023). These requirements act as sequential “filters”, meaning that most peptides identified from the sequencing data fail at some of these stages (Figure 2).

Computational pipelines, now increasingly AI-enabled, address this challenge by navigating this complex landscape, with the ultimate goal of narrowing down the tumor-derived peptides that are most likely to represent viable neoantigen vaccine candidates and are therefore suitable for downstream validation experiments, which often include HLA binding confirmation and immunological analysis that assess the presence of existing T-cells able to recognize the selected peptide (Wells et al., 2020).

### 2.1 End-to-end computational pipeline

A typical computational workflow for neoantigen prediction is based on the tumor next-generation sequencing (NGS) data. It is used to identify the mutations present in the tumor tissue, filter them, and predict the peptide’s HLA binding and T-cell receptor recognition potential (Fotakis et al., 2021).

#### 2.1.1 Tumor sequencing (WES/WGS/RNA-seq)

To identify tumor mutations that produce peptide candidates, both tumor and healthy tissue (usually blood) is sequenced. Usually, whole-exome sequencing (WES) is performed on the samples, because it specifically targets protein-coding regions. Some pipelines go further, and rely on whole-genome sequencing, which can more easily capture more changes, including structural variants, gene fusions, and non-coding alterations. RNA sequencing can be added to estimate the abundance of aberrant peptides, on the basis of RNA expression data (Zhao et al., 2025). This data is also used to perform HLA typing, which is the basis of predicting the peptide-HLA binding potential.

#### 2.1.2 Mutation calling and filtering

After NGS data preprocessing and quality control, genetic variants are called (identified) by comparing the sequencing data to a public reference genome. At this stage, the goal is to detect changes such as single-nucleotide variants (SNVs), insertions and deletions (indels), gene fusions, larger structural variants, and splice-altering mutations. Variant calling is most commonly performed using tools such as HaplotypeCaller (GATK (Van der Auwera and O’Connor)), DRAGEN (DRAGEN secondary analysis (4.3). Illumina, 2024), DeepVariant (Poplin et al., 2018), FreeBayes (Garrison and Marth, 2012) for germline data, and Mutect2 (GATK (Van der Auwera and O’Connor, 2026)), DRAGEN (DRAGEN secondary analysis (4.3). Illumina, 2024), DeepSomatic (Park et al., 2025), Strelka2 (Kim et al., 2018), VarScan2 (Koboldt et al., 2012) for somatic tumor data. Also, Manta (Chen et al., 2016), Delly (Rausch et al., 2012) for structural variants and gene fusions. Sequencing both tumor and matched normal (healthy) samples is essential to distinguish somatic mutations from germline variants, thereby reducing the risk of selecting targets that could induce autoimmunity. In addition, not all somatic mutations are equally relevant. Subclonal mutations (present in only a small fraction of tumor cells) may be insufficient to elicit a strong immune response. Similarly, mutations that are not supported by RNA sequencing data may indicate low or absent expression, suggesting that the corresponding peptides are unlikely to be produced at levels sufficient for effective antigen presentation.

#### 2.1.3 Neoantigen identification and prioritization

To finally identify and prioritize neoantigens, different tools are used to predict the peptide-HLA binding (NetMHCpan (Reynisson et al., 2020), NetMHCIIpan (Reynisson et al., 2020), MHCflurry (O'Donnell et al., 2020), MixMHCpred (Tadros et al., 2025)) and T-cell receptor recognition potential (NetTCR (Jensen and Nielsen, 2024), MixTCRpred (GfellerLab/MixTCRpred, 2025), TCR-ESM (Yadav et al., 2023)). At the pipeline level, the whole process of neoantigen prioritization can be streamlined with tools such as pVACtools (Hundal et al., 2020), MuPeXi (Bjerregaard et al., 2017), and VaxRank (Rubinsteyn et al., 2018)

### 2.2 Machine learning and deep learning approaches

#### 2.2.1 Conventional supervised vs. deep neural models

In supervised learning, models are trained on labeled datasets containing known outcomes, for example, - whether a given peptide binds to a specific HLA molecule. Based on this training data, the models learn patterns that enable them to predict binding or non-binding for previously unseen peptide-HLA pairs. Traditional machine learning approaches, such as support vector machines and random forests, have been widely used in early epitope prediction tasks. However, the emergence of deep learning has significantly advanced the field. Architectures such as convolutional neural networks (CNNs), recurrent neural networks (e.g., LSTM), transformers, attention-based models, and graph neural networks (GNNs) provide greater flexibility and capacity to model complex, non-linear relationships in high-dimensional and multimodal biological data. These capabilities are particularly important in neoantigen prediction, where multiple factors, such as peptide sequence, HLA type, and gene expression, interact in a highly complex manner. Deep learning models excel at capturing such interactions and have therefore become the dominant approach in modern pipelines.

#### 2.2.2 Training data (immunopeptidomics, public databases)

The development of AI models for neoantigen prediction relies on several key public databases. Somatic variant calling is supported by databases such as dbSNP (Sherry et al., 2001) and the 1000 Genomes Project (Fairley et al., 2020), which provide commonly observed human genetic variation, and the COSMIC (Tate et al., 2019) database, which provides curated somatic mutations to support prioritization of cancer-relevant variants (Zhao et al., 2025).

The most prominent database providing the peptide-HLA interaction information is The Immune Epitope Database (IEDB) (Vita et al., 2025) which contains experimental data on antibody and T-cell epitopes studied in humans and other animal species. Its companion site, CEDAR (Koşaloğlu-Yalçın et al., 2023), contains data on epitopes studied in context of cancer disease.

Immunopeptidomics is an emerging branch of proteomics that studies peptides naturally presented on MHC molecules in cells. Using mass spectrometry, these peptides can be directly identified from biological samples, rather than inferred from *in vitro* binding assays. This is important because it confirms that peptides are not only capable of binding MHC, but are also properly produced, processed, and presented in a physiological context. As a result, immunopeptidomics provides high-quality data that is increasingly used to improve neoantigen prediction, particularly in AI-based models.

### 2.3 Advantages of AI over rule-based prediction

Traditionally, rule-based methods were used to predict T-cell epitopes, relying on known binding motifs and peptide patterns, but they often fail to generalize to novel alleles or unconventional epitopes. In contrast, deep neural networks can capture complex, non-linear relationships between amino acid features and immunogenicity (Villanueva-Flores et al., 2025).

### 2.4 Current performance limits and false-positive burden

The Tumor Neoantigen Selection Alliance (TESLA) is a global initiative aimed at improving the understanding of tumor epitope immunogenicity, refining neoantigen prediction methods, and establishing standardized benchmarking datasets (Wells et al., 2020). In a TESLA study, only 37 out of 608 (6%) candidate peptides were confirmed to be immunogenic, highlighting the substantial false-positive burden that persists despite recent advances in AI-based approaches.

The biological causes of this gap are increasingly well characterized and explain why purely architectural improvements to AI models have produced diminishing returns. First, training data remain incomplete: immunopeptidomics datasets, despite their growth, sample only a fraction of HLA alleles and tissue contexts, so models extrapolate beyond their training distribution when applied to novel patient backgrounds (Conev et al., 2023). Second, antigen processing is context-dependent - proteasome composition (constitutive vs. immunoproteasome), inflammatory state, and cell-type-specific protease repertoires alter which peptides are generated from a given parent protein, yet most predictors treat processing as a single fixed function (Zou et al., 2025). Third, a growing fraction of validated tumor antigens derives from non-canonical translation products, including peptides from cryptic open reading frames, retained introns, untranslated regions, and long non-coding RNAs; pipelines built on canonical proteome annotations systematically miss these “dark” antigens and may rank truly immunogenic candidates below conventional but ultimately non-productive ones (Ouspenskaia et al., 2022). Fourth, peptide–HLA complex stability, the off-rate of the bound peptide, predicts immunogenicity better than affinity alone, because durable presentation is required for T-cell priming, yet most predictors output affinity rather than stability and the two are only loosely correlated (Harndahl et al., 2012). Together, these factors imply that closing the validation gap will require expanded and more diverse training data, explicit modeling of processing context, incorporation of non-canonical antigen sources, and a shift from affinity-centric to stability-aware presentation models.

## 3 AI for HLA binding, antigen processing, and immunogenicity prediction

Building on the discovery and prioritization steps outlined above, this section moves downstream along the cascade, from the upstream filters of expression and variant calling to the central biological gateways of HLA binding, antigen processing, presentation, and T-cell recognition. Each filter narrows the candidate pool further, and each is modeled by AI with markedly different levels of maturity and reliability. Table 1 summarizes representative AI tools spanning the two filters where prediction is most actively modeled (peptide–HLA binding/presentation and TCR–peptide recognition) together with their architectures, input features, and current level of experimental or clinical validation.

**TABLE 1:** Comparison of AI tools across the peptide–HLA and TCR–peptide prediction axes.

Tool (ref)	Axis/task	Architecture	Key inputs	Training data	Validation status
NetMHCpan-4.1 (Reynisson et al., 2020)	Peptide-HLA-I binding + presentation	Pan-allelic shallow NN ensemble (NNAlign_MA)	Peptide + HLA pseudo-sequence	Binding affinity + MS eluted-ligand	Extensively benchmarked; embedded in trial pipelines
NetMHCIIpan-4.0 (Reynisson et al., 2020)	Peptide-HLA-II binding	Pan-allelic NNAlign	Peptide + HLA-II sequence	Affinity + eluted-ligand	Benchmarked; used for CD4 epitope selection
MHCflurry 2.0 (O'Donnell et al., 2020)	Peptide-HLA-I presentation	Feed-forward NN ensemble + antigen-processing predictor	Peptide + allele	Affinity + MS ligands	Benchmarked; open-source; used in pipelines
MixMHCpred (Tadros et al., 2025)	Peptide-HLA-I/II (motif)	Motif deconvolution/PWM	Peptide + allele	MS immunopeptidomics	Benchmarked; academic-pipeline use
NetTCR-2.x (Jensen and Nielsen, 2024)	TCR-peptide recognition	CNN	Paired α/β CDR3 + peptide	VDJdb/IEDB pairs	Top-tier on seen epitopes; near-random on unseen (Lu et al., 2026)
MixTCRpred (GfellerLab/MixTCRpred, 2025)	TCR-epitope recognition	Transformer	CDR3α/β + epitope	Curated pairs	Effective on seen epitopes; not clinically validated (Lu et al., 2026)
TCR-ESM (Yadav et al., 2023)	TCR-peptide-MHC	Protein-LM (ESM) embeddings	TCR + peptide + MHC sequence	IEDB/VDJdb	Research-only benchmarking
epiTCR (Pham et al., 2023)	TCR-peptide binding	Random forest (BLOSUM62 encoding)	CDR3β + peptide	>3M TCR-peptide pairs (5 public DBs)	High sensitivity on seen epitopes; degrades on unseen (Lu et al., 2026)

Abbreviations: NN, neural network; CNN, convolutional neural network; PWM, position weight matrix; MS, mass spectrometry; LM, language model. Validation status reflects the highest level reached: retrospective benchmarking, use in research pipelines, or embedding in clinical-trial pipelines. The collapse of all TCR–peptide predictors toward random performance on unseen epitopes is established by the 50-model benchmark of Lu et al. (Lu et al., 2026).

### 3.1 AI-based prediction of peptide–HLA binding

A central step in peptide cancer vaccine design is identifying peptides capable of binding to HLA molecules with sufficient affinity to be presented on the cell surface (Kessler et al., 2025). Although these two events are frequently conflated in computational and clinical literature, binding affinity is necessary but not sufficient for presentation; following the cascade framing introduced above, we treat them as separate filters with distinct AI-modeling histories and discuss antigen processing and presentation in Section 3.2. The extreme polymorphism of HLA genes presents a major computational challenge, as each allele exhibits distinct peptide-binding preferences. Early prediction methods relied on motif-based approaches and position-specific scoring matrices, which were limited in their ability to generalize across alleles and capture complex sequence dependencies.

The introduction of artificial intelligence, particularly machine learning, significantly improved predictive performance. Supervised learning models such as gradient boosting machines and shallow neural networks enabled the integration of diverse sequence features, capturing non-linear relationships between peptide composition and binding affinity (Yin et al., 2024). More recently, deep learning architectures, including convolutional neural networks and transformer-based models, have further advanced the field by enabling end-to-end sequence modeling. Modern AI-based predictors often adopt pan-allelic frameworks, encoding both peptide and HLA sequences to allow predictions across a wide range of alleles, including rare or poorly characterized variants. This approach is particularly important for addressing the global diversity of HLA genotypes and improving the inclusivity of vaccine design strategies. Training datasets derived from large-scale binding assays and immunopeptidomics experiments have been instrumental in enabling these advances (Vo et al., 2025).

### 3.2 Modeling antigen processing and presentation pathways

While peptide–HLA binding is necessary for antigen presentation, it is not sufficient. The generation of presented peptides is governed by a multi-step intracellular process involving proteasomal cleavage, transport via the transporter associated with antigen processing (TAP), and loading onto HLA molecules within the endoplasmic reticulum (Mantel et al., 2022). Each of these steps imposes additional constraints on which peptides ultimately reach the cell surface.

AI has increasingly been applied to model these upstream processes. Machine learning models trained on proteasomal cleavage data can predict the likelihood that a given peptide will be generated from a parent protein (Villanueva-Flores et al., 2025). Similarly, models estimating TAP transport efficiency provide insight into whether peptides can be effectively translocated into the endoplasmic reticulum. Integrative frameworks combine predictions from these individual steps with HLA binding affinity to generate a more comprehensive estimate of presentation probability.

The emergence of immunopeptidomics has been particularly transformative in this context. Mass spectrometry-based datasets provide direct evidence of peptides naturally presented on HLA molecules *in vivo*. AI models trained on such data implicitly capture the combined effects of antigen processing and presentation, often outperforming models based solely on binding affinity (Vo et al., 2025). These approaches represent a shift from theoretical prediction toward empirically grounded modeling of antigen presentation.

### 3.3 AI approaches to immunogenicity prediction

The transition from presentation to recognition marks the point in the cascade at which AI predictive performance deteriorates most sharply. A critical challenge in peptide vaccine development is distinguishing between peptides that are merely presented and those that elicit a functional T-cell response (Malonis et al., 2020). Binding affinity and presentation likelihood do not necessarily correlate with immunogenicity, as effective immune activation depends on recognition by T-cell receptors (TCRs) and subsequent signaling cascades.

AI-based immunogenicity prediction models attempt to address this gap by incorporating additional biological features (Kong, 2025). These include sequence similarity to known immunogenic epitopes, physicochemical properties of peptides, and inferred structural characteristics of the peptide–HLA complex. Some models also integrate experimental data on T-cell responses, although such datasets remain relatively limited compared to binding data (Chao et al., 2025). More advanced approaches aim to model peptide–HLA–TCR interactions directly (Cao et al., 2026). These methods often leverage deep learning architectures and structural modeling techniques to approximate the likelihood of TCR engagement. However, the scarcity of high-quality paired peptide–HLA–TCR datasets continues to constrain progress in this area. As a result, immunogenicity prediction remains one of the most challenging aspects of AI-driven vaccine design.

### 3.4 Immunodominance and multi-modal AI models

Not all presented epitopes contribute equally to the immune response. The phenomenon of immunodominance, whereby a limited subset of epitopes elicits a robust response, adds another layer of complexity to vaccine design (Graham et al., 2018). Predicting which peptides will become immunodominant requires consideration of factors beyond sequence and binding affinity, including antigen abundance, competition between epitopes for limited TCR repertoire space, and the kinetics of antigen-presenting cell engagement.

AI models are increasingly incorporating multi-modal data to address this challenge (Schuhmacher et al., 2026). Features such as gene expression levels, peptide abundance, tumor heterogeneity, and characteristics of the tumor microenvironment can all influence epitope visibility and immune activation. Integrating genomic, transcriptomic, and proteomic data allows models to better approximate the biological context in which antigen presentation occurs (Vo et al., 2025). However, the architectures used for this integration carry distinct limitations that are rarely interrogated in vaccine-focused literature. Transformer-based predictors and protein foundation models inherit the distributional biases of their pretraining corpora, which are dominated by UniProt-derived canonical proteomes rather than immunopeptidomes; this constrains their ability to model the non-canonical peptide space discussed in Section 2.4. Graph-based approaches that explicitly represent peptide–HLA–TCR interactions face a cardinality mismatch between the small set of characterized TCRs in public databases and the orders-of-magnitude larger space of patient repertoires. Multimodal fusion is itself unstable on the small cohorts typical in oncology, where late fusion frequently outperforms early fusion, not because it captures biology more faithfully but because it is more statistically identifiable with limited samples (Stahlschmidt et al., 2022).

A further conceptual issue concerns the distinction between predictive correlation and mechanistic causality. Multimodal models that combine expression, mutation, immunopeptidomic, and microenvironment features can improve prediction of immunogenicity outcomes on held-out test sets, but the improvements are typically driven by correlative associations rather than by representations of the underlying biology. As a consequence, model performance often degrades when applied to cohorts outside the training distribution, including different cancer types, treatment contexts, or HLA backgrounds, because the learned associations do not transfer with the same fidelity as causal mechanisms would. This distinction matters clinically: an enrichment tool whose ranking reflects correlation can still accelerate candidate selection, but it cannot reliably explain why a given patient failed to respond, nor can it be safely extrapolated to underrepresented populations without re-validation.

These multi-modal approaches nevertheless represent a meaningful shift toward systems-level modeling of tumor–immune interactions. Their near-term value lies in narrowing experimental search spaces and prioritizing candidates for empirical validation, rather than in serving as standalone selection criteria. Realizing their full potential will require larger and more representative training cohorts, explicit modeling of patient-specific TCR repertoires, and architectures that incorporate mechanistic priors from immunology rather than relying solely on end-to-end learning.

### 3.5 Population diversity, bias, and model limitations

The high degree of HLA polymorphism across human populations presents a significant challenge for both personalized and population-level vaccine strategies. AI-driven pan-allelic models partially address this issue by enabling predictions across diverse HLA types. Additionally, population-level analyses can identify peptides with high predicted binding affinity across multiple common alleles, supporting the development of broadly applicable vaccines. However, biases in training datasets remain a concern (Gote, 2026). Many datasets are skewed toward well-characterized HLA alleles that are more prevalent in European populations, potentially limiting the generalizability of AI models to underrepresented groups. Addressing this imbalance will require more diverse data collection and targeted model development. Despite substantial progress, AI predictions are not infallible. False positives remain common, and predicted epitopes do not always translate into *in vivo* immunogenicity (Vo et al., 2025). Consequently, experimental validation remains essential. Efforts to standardize benchmarking datasets and evaluation metrics are ongoing, aiming to improve reproducibility and facilitate comparison across different models.

### 3.6 Biological determinants constraining AI generalizability

The persistent gap between computational prediction and experimental validation reflects biological constraints that operate at and between filters of the cascade, and that current AI architectures capture only partially or not at all. Upstream of presentation, central tolerance shapes which neoantigens can ever be recognized: AIRE-driven expression of tissue-restricted antigens in medullary thymic epithelial cells deletes self-reactive clones during thymic selection, and neoantigens whose mutated residues lie outside the TCR contact interface, or that closely resemble such self-peptides, may encounter a functionally depleted repertoire (Anderson and Su, 2016). Even when a cognate clone survives selection, it must also be present in the individual patient’s naive repertoire at the time of vaccination—a repertoire shaped by age, prior infections, chemotherapy, and lymphodepletion that sequence-based predictors do not access (Schumacher and Schreiber, 2015). Static binding-affinity and presentation models compound this limitation by treating the peptide–HLA complex as a single low-energy structure, whereas the TCR engages a conformational ensemble in which adequate binding does not guarantee a TCR-competent conformer. Presentation itself is not binary: below threshold copy number, presented peptides can elicit anergy rather than activation, and above threshold they compete for finite CD8 expansion capacity, producing the immunodominance hierarchies discussed in Section 3.4.

Downstream of presentation, tumor biology further constrains what AI predictions can deliver. Tumors that reach clinical attention have already undergone immunoediting (Schreiber et al., 2011); the antigenic repertoire visible at biopsy is enriched for epitopes that endogenous immunity failed to eliminate, so vaccination strategies prioritizing the highest-ranked predicted neoantigens may systematically target what evolution has already declared safe. Spatially and temporally, the antigenic landscape diverges across lesions and across the weeks between biopsy and vaccine administration: clonal neoantigens shared by all tumor cells are most predictive of immunotherapy response, but subclonal antigens dominate many AI candidate lists (McGranahan et al., 2016). Finally, even valid vaccine-induced responses can be blunted within the tumor microenvironment by PD-1/PD-L1 engagement, regulatory T-cell infiltration, metabolic restriction, and TGF-β signaling, contributing to the recurring disconnect between vaccine-induced peripheral immune responses and meaningful clinical benefit (Philip and Schietinger, 2022). Collectively, these determinants suggest that the validation gap exposed by TESLA is not primarily an algorithmic problem to be solved by larger models, but a data and biology problem. Until training data captures patient-specific repertoires, abundance-calibrated presentation, and immunoediting priors, AI predictions should be understood as enrichment tools rather than as definitive selection criteria.

## 4 Personalization, clinical translation, and current evidence

At the end of the cascade, computational predictions must translate into clinically meaningful immune responses in individual patients, the stage at which the cumulative attrition of upstream filters becomes most visible. A major shift in cancer vaccine development has been the transition toward personalized neoantigen-based approaches. Unlike vaccines targeting shared tumor-associated antigens, these strategies are tailored to the individual mutational landscape of each patient’s tumor. High-throughput sequencing enables the identification of somatic mutations, while computational pipelines predict which mutated peptides are likely to bind patient-specific HLA molecules and elicit T-cell responses (Zeng et al., 2026). Personalized neoantigen vaccines offer several advantages. Because neoantigens are restricted to tumor cells, the risk of off-target autoimmune toxicity is reduced. Their non-self origin also increases the likelihood of strong immunogenicity (Xie et al., 2023). However, this approach introduces substantial logistical complexity, as each vaccine must be designed, manufactured and delivered on a per-patient basis. Artificial intelligence is increasingly applied to address these challenges. Machine learning models trained on large peptide–MHC datasets can predict binding affinity and complex stability with increasing accuracy (Elfatimi et al., 2025). More advanced approaches integrate additional biological parameters, including antigen processing, transcript abundance, peptide presentation and T-cell receptor recognition, to prioritize the most relevant epitopes (Shah et al., 2023). This reduces the experimental burden and accelerates candidate selection. Beyond antigen prioritization, computational approaches also enable patient stratification. Integrated models can analyze genomic alterations, tumor mutational burden, immune infiltration and HLA genotype to identify patients most likely to benefit from vaccination (Alshorman et al., 2026; Sun et al., 2025). Such stratification is critical given the variability in tumor immunogenicity across patients and cancer types. However, the clinical performance of AI-driven predictions remains limited by data quality, incomplete modeling of antigen processing and variability in T-cell receptor recognition.

Early clinical data support the feasibility and immunogenicity of neoantigen vaccines. Studies in melanoma have shown that personalized peptide- and RNA-based vaccines can induce robust CD4-positive and CD8-positive T-cell responses targeting multiple tumor-specific mutations (Jahantigh et al., 2026; Wu et al., 2025; Ott et al., 2017). These responses are often durable, and in some cases are associated with prolonged progression-free survival. Importantly, emerging data from glioblastoma further support the clinical potential of this approach. In a cohort of 173 patients treated with personalized neoantigen peptide vaccines, median overall survival reached 31.9 months, with significantly prolonged survival observed in patients mounting multiple vaccine-induced T-cell responses. Vaccine-specific immune responses were detected in 90% of monitored patients and were durable in the majority of cases, while adverse events remained predominantly mild (Latzer et al., 2024). These findings indicate that personalized neoantigen vaccines can induce robust and clinically meaningful immune responses even in highly aggressive malignancies. In addition to their immunogenicity and emerging clinical efficacy, the safety profile of neoantigen vaccines has been consistently favorable. Table 2 summarizes representative neoantigen vaccine programs, the computational pipelines they employ, and their clinical status and outcomes.

**TABLE 2:** Computational pipelines in representative neoantigen vaccine programs and trials.

Program/trial (NCT/ref)	Cancer	Platform	Pipeline/tools	Phase/status	Key outcome
KEYNOTE-942/mRNA-4157 (intismeran autogene) (NCT03897881) (Weber et al., 2024)	Melanoma	mRNA (≤34 neoAg)	Proprietary (Moderna; tools not disclosed)	Ph 2b (n = 157) → Ph 3	RFS HR 0.561 (95% CI 0.309–1.017); 18-mo RFS 79% vs. 62%
Autogene cevumeran/BNT122 (NCT04161755) (Rojas et al., 2023)	PDAC → melanoma, CRC	mRNA-lipoplex (≤20 neoAg)	Proprietary (BioNTech; not disclosed)	Ph 1 (n = 16) → Ph 2	8/16 responders; RFS not reached vs. 13.4 mo
NeoVax (NCT01970358) (Ott et al., 2017)	Melanoma	Peptide (SLP)	NetMHCpan-based selection	Ph 1 (n = 6)	Safe, immunogenic; 4/6 recurrence-free at 25 mo
PGV-001 (Saxena et al., 2025)	Multiple solid	Peptide (SLP)	Open: Isovar, VaxRank, NetMHCpan + RNA abundance	Ph 1	Feasible, immunogenic
WashU program (multi-NCT) (Singhal et al., 2025)	Multiple	Peptide/DC	Open: pVACtools (pVACseq/pVACfuse)	∼185 pts/11 trials	Ongoing
GBM cohort (Latzer et al., 2024)	Glioblastoma	Peptide	Per source (e.g., OptiType HLA typing)	Real-world cohort (n = 173)	Median OS 31.9 mo; 90% immune response

Abbreviations: neoAg, neoantigens; SLP, synthetic long peptide; PDAC, pancreatic ductal adenocarcinoma; CRC, colorectal cancer; DC, dendritic-cell; RFS, recurrence-free survival; OS, overall survival. Pivotal positive trials rely on undisclosed proprietary pipelines, whereas reproducible open-source tools dominate academic programs; no trial pipeline yet incorporates a TCR-recognition predictor.

Most adverse events are mild to moderate and include injection-site reactions and transient flu-like symptoms. Because peptide-based vaccines do not rely on replicating systems or viral vectors, the risk of severe systemic toxicity remains low (Wang et al., 2026). Nonetheless, long-term safety monitoring remains necessary, particularly in combination regimens. Combination strategies are likely to be critical for clinical efficacy. Immune checkpoint pathways such as PD-1 and CTLA-4 suppress T-cell activity within the tumor microenvironment, limiting the effectiveness of endogenous and vaccine-induced responses (Vlachostergios, 2025). Checkpoint inhibition can restore T-cell function and enhance the activity of vaccine-primed lymphocytes. Early clinical observations suggest that vaccines may broaden the repertoire of tumor-specific T-cells, while checkpoint inhibitors sustain their effector function, resulting in a complementary therapeutic effect (Chen H. et al., 2020; Wang et al., 2026).

Despite this progress, several translational challenges remain, and the field’s enthusiasm has at times outpaced the strength of the underlying evidence base. The most immediate constraint is the data foundation on which AI prediction is built. Training datasets for peptide–HLA binding and presentation are heavily skewed toward HLA alleles prevalent in European-ancestry populations, with HLA-A*02:01 alone disproportionately represented across IEDB, mass-spectrometry datasets, and the training sets of widely used predictors; benchmarking on alleles common in African, Asian, and Indigenous populations consistently shows degraded performance (Conev et al., 2023). Reproducibility across laboratories and pipelines is also limited: different combinations of variant callers, HLA typers, binding predictors, and prioritization thresholds applied to the same sequencing data routinely produce non-overlapping candidate lists (Fotakis et al., 2021; Zhao et al., 2025). Prospective, head-to-head benchmarking of competing AI pipelines within actual vaccine trials remains rare (Ott, 2026), which means that improvements demonstrated on retrospective TESLA-like benchmarks have not been independently confirmed in clinical decision-making contexts. Until external validation across populations and prospective benchmarking become standard, claims of pipeline superiority should be treated cautiously.

Manufacturing and regulatory considerations present additional barriers. Turnaround time from tumor sequencing to neoantigen prediction, peptide synthesis, and vaccine formulation often spans several weeks, which limits applicability in aggressive disease (Ott, 2026; Truex et al., 2020). Personalized vaccines require flexible, small-scale production systems under stringent quality control, increasing cost and constraining scalability. Existing regulatory frameworks are not fully adapted to individualized therapies, and a further layer of uncertainty surrounds AI systems that update after deployment: regulators have begun to distinguish locked from adaptive algorithms, but consensus standards for when retraining requires re-review, how performance drift should be monitored, and how to validate predictions that change between patients have not yet stabilized (Ott, 2026; Skerritt, 2025). Balancing safety, reproducibility, and the inherent flexibility of personalized AI-driven design remains a key implementation challenge.

A more fundamental concern is the recurring disconnect between vaccine-induced immunogenicity and meaningful clinical benefit. Across multiple trials of personalized neoantigen vaccines, robust antigen-specific T-cell responses have been documented in peripheral blood without commensurate improvements in progression-free or overall survival (Ott, 2026). The reasons span the biological determinants discussed in Section 3.6: tumor-microenvironment-imposed exhaustion, immunoediting-driven antigen loss, spatiotemporal heterogeneity of the antigenic landscape, and the gap between peripheral T-cell expansion and effective intratumoral killing. Encouraging signals such as the glioblastoma cohort discussed above demonstrate that this disconnect is not inevitable, but they remain exceptions rather than the rule, and the field has not yet established which patients, tumor types, or combination regimens reliably convert vaccine-induced immunity into durable clinical benefit. Addressing this gap will require coordinated efforts across disciplines, including continued advances in AI-driven prediction, automated manufacturing, adaptive regulatory strategies, and trial designs that explicitly measure intratumoral immune function alongside peripheral responses. Taken together, these considerations indicate that personalized cancer vaccines are approaching clinical viability but remain at an earlier translational stage than the most optimistic accounts suggest.

Translating these advances into routine practice will require closing the validation gap prospectively rather than retrospectively. The tools summarized here have largely been benchmarked on historical datasets, and their clinical value now depends on prospective testing against standardized, independently curated benchmarks of the kind TESLA was designed to provide (Wells et al., 2020), so that reported gains reflect genuine immunogenicity rather than dataset-specific overfitting. In parallel, reliable identification of the patients most likely to respond—through integrated models combining tumor mutational burden, HLA genotype, and immune-microenvironment features—will be needed to translate population-level accuracy into individual benefit. Scalability poses a further, distinct barrier: because each vaccine must be designed, manufactured, and released within a clinically actionable window, the “vein-to-vein” interval and the regulatory frameworks governing patient-specific biologics will strongly influence how widely these therapies can be deployed (Truex et al., 2020; Skerritt, 2025). Notably, the two pivotal positive trials rely on undisclosed, proprietary prediction pipelines (Table 2), highlighting a tension between commercial development and the reproducibility and regulatory transparency that broad clinical adoption ultimately requires. In the nearer term, AI-guided design of shared-antigen, “off-the-shelf” vaccines may provide a more scalable complement to fully personalized regimens, extending neoantigen-directed immunotherapy beyond centers equipped for individualized manufacturing.

## 5 Conclusion

Artificial intelligence is increasingly being integrated across peptide-based cancer vaccine development, from antigen discovery and epitope prediction to patient stratification and clinical trial design. By enabling the integration and large-scale analysis of complex genomic and immunological data, AI improves the identification of clinically relevant neoantigens and accelerates vaccine development. These advances directly address key limitations that have historically constrained peptide-based strategies, particularly in antigen selection and prioritization. However, computational prediction alone is insufficient. Experimental validation of antigen processing, MHC presentation and T-cell recognition remains essential to establish biological and clinical relevance. The integration of AI-driven approaches with rigorous immunological validation will therefore be critical for successful clinical translation. Future progress will depend on combination strategies that integrate personalized vaccines with immune checkpoint inhibitors and other immunomodulatory therapies. In this context, AI is expected to play a central role in optimizing therapeutic design, enabling adaptive and patient-specific treatment approaches. Continued advances in data integration, model robustness and clinical implementation will be required to fully realize the potential of AI-guided cancer vaccines in precision oncology.

## 6 Key terms

Neural Network - A model of layered, interconnected units whose weights are tuned on data to map an input (e.g., a peptide sequence) to an output (e.g., a binding score).

Transformer - A neural-network architecture that weighs relationships among all positions in a sequence at once (“attention”), well suited to peptide, HLA, and TCR sequences.

Pan-allelic model - A predictor trained across many HLA alleles at once, allowing it to score peptides even for alleles with little or no experimental data.

Immunopeptidomics - Mass-spectrometry identification of the peptides actually presented on HLA molecules, giving “ground-truth” presentation data for training and validation.

Eluted-ligand data - Peptides experimentally shown to be HLA-bound (from immunopeptidomics), used alongside binding-affinity data to train presentation predictors.

Foundation model - A large model pretrained on broad biological sequence data, then adapted to specific tasks such as epitope or TCR prediction.

Validation gap - The persistent discrepancy between peptides predicted to be immunogenic and those experimentally confirmed to elicit T-cell responses (≈6% confirmed in TESLA).
